# How I do it: parietal trans-sulcal para-fascicular approach to lateral thalamic/internal capsule cavernous malformation

**DOI:** 10.1007/s00701-021-04884-2

**Published:** 2021-06-24

**Authors:** Michael Amoo, Kieron J. Sweeney, Ronan Kilbride, Mohsen Javadpour

**Affiliations:** 1 Department of Neurosurgery, Beacon Hospital, Sandyford, Dublin 18, Ireland; 2 National Neurosurgical Centre, Beaumont Hospital, Dublin 9, Ireland; 3 Royal College of Surgeons in Ireland, Dublin, Ireland; 4 Department of Neurology and Clinical Neurophysiology, Beaumont Hospital, Dublin 9, Ireland; 5 Department of Academic Neurology, Trinity College Dublin, Dublin, Ireland

**Keywords:** Thalamus, Internal capsule, BrainPath®, Tubular retractor, Para-fascicular surgery, Cavernous malformation, Minimally invasive neurosurgery, Exoscope, ORBEYE®

## Abstract

**Background:**

The surgical management of deep brain lesions is challenging, with significant morbidity. Advances in surgical technology have presented the opportunity to tackle these lesions.

**Methods:**

We performed a complete resection of a thalamic/internal capsule CM using a tubular retractor system via a parietal trans-sulcal para-fascicular (PTPF) approach without collateral injury to the nearby white matter tracts.

**Conclusion:**

PTPF approach to lateral thalamic/internal capsule lesions can be safely performed without injury to eloquent white matter fibres. The paucity of major vessels along this trajectory and the preservation of lateral ventricle integrity make this approach a feasible alternative to traditional approaches.

**Supplementary Information:**

The online version contains supplementary material available at 10.1007/s00701-021-04884-2.

## Introduction and relevant anatomy

**Fig. 1:**
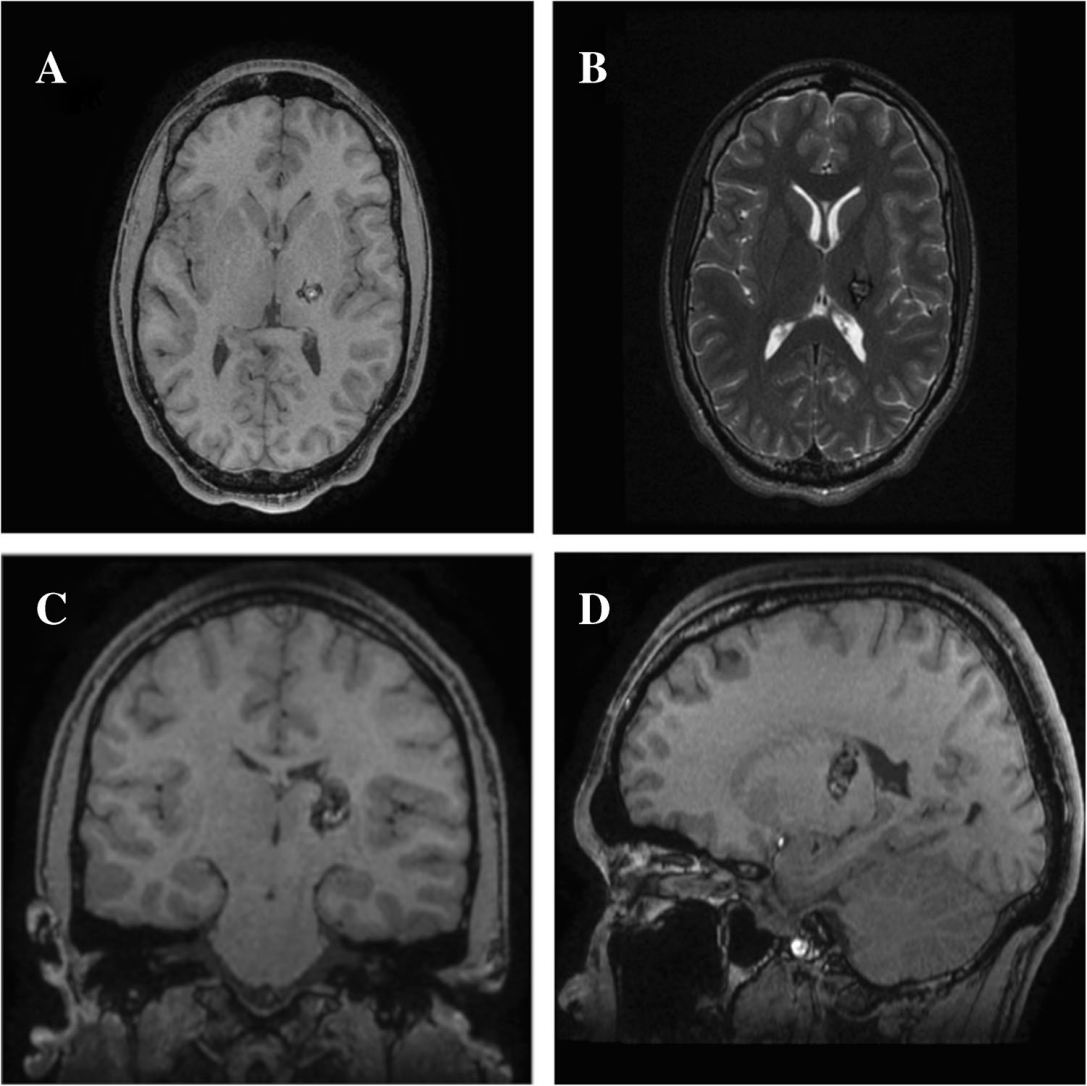
Preoperative non-contrast magnetic resonance imaging showing a hyperintense lesion within a hypointense haemosiderin ring in the left lateral thalamus, consistent with a cavernoma (**A** axial T1-weighted; **B** axial T2 weighted; **C** coronal reconstruction of T2 weighted: and **D** sagittal reconstruction of T1-weighted images)

**Fig. 2:**
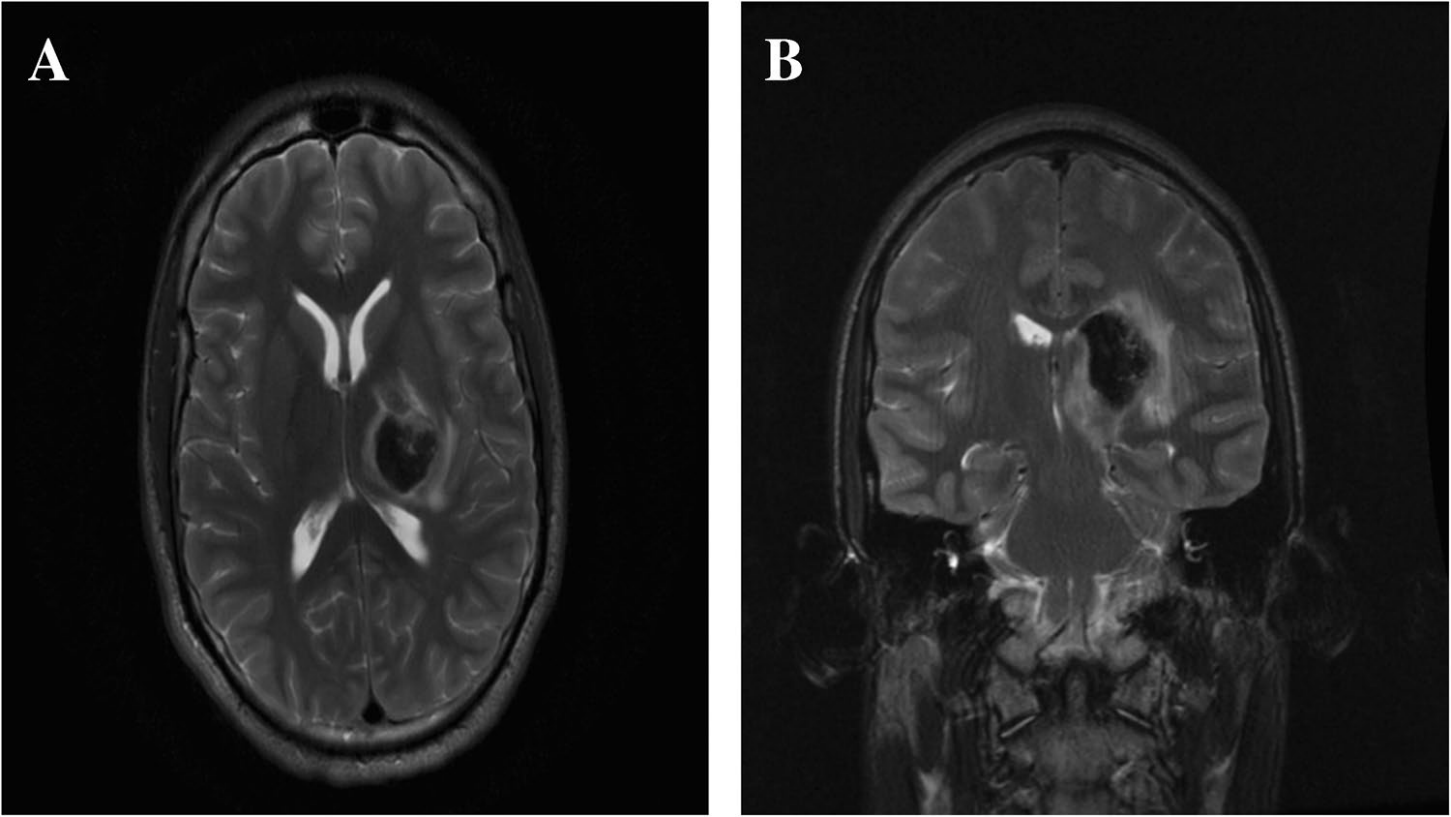
Magnetic resonance imaging (**A** axial; **B** coronal) demonstrating a large left thalamic haemorrhage

**Fig. 3:**
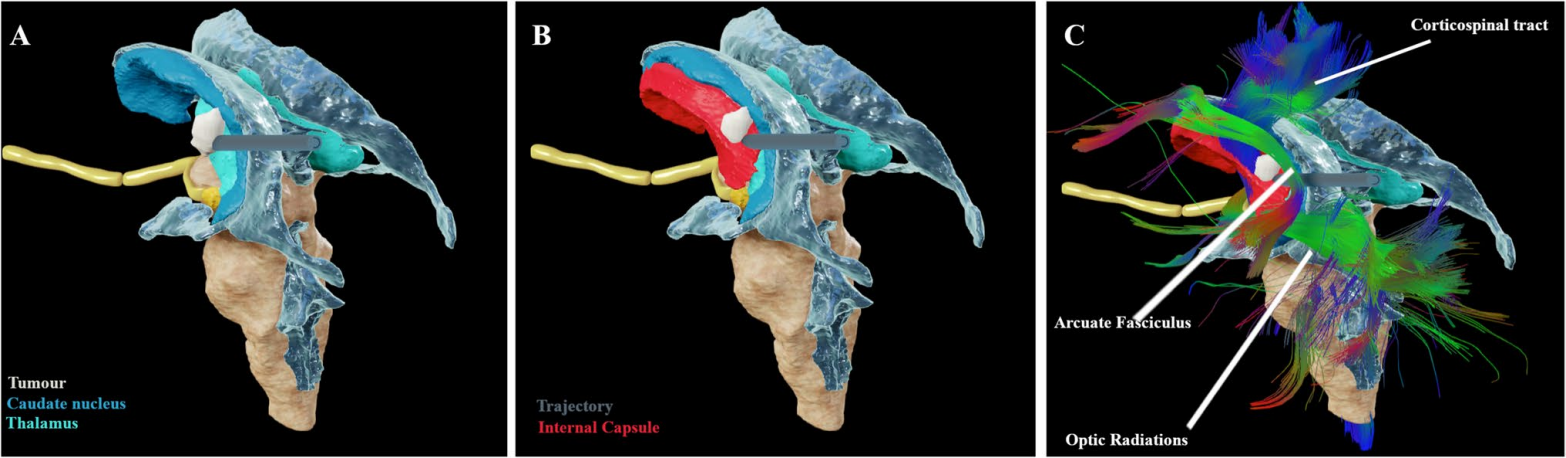
Magnetic resonance and diffusion tensor images, reconstructed and segmented in Brainlab Elements, illustrating the operative trajectory in relation to surrounding critical structures. **A** Model illustrating the cavernous malformation within lateral thalamus; **B** internal capsule (red) added; **C** corticospinal tract (anterior and lateral to cavernoma), arcuate fasciculus, and optic radiations projected

Cavernous malformations (CM) involving deep structures, such as basal ganglia and thalamus, account for roughly 5–10% of cerebral CM [3, 8]. The eloquence of this region restricts microsurgery to narrow operative corridors. The thalamus is a gateway for major sensory pathways to the cerebral cortex. Additionally, it is involved in connections with the extra-pyramidal motor, visual, auditory, limbic, and consciousness systems. It is divided into various specific and non-specific functional nuclei, and closely related, anatomically, to the lateral and third ventricles, caudate nucleus, and the genu and posterior limb of the internal capsule (IC). Therefore, haemorrhage or surgical manipulations in this vicinity could result in a range of neurological deficits, i.e. hemi-paresis or hemi-sensory loss, visual impairment, and disorders of consciousness and memory. We present the case of a 23-year-old right-handed gentleman with a left postero-lateral thalamus CM (Fig. 1). He previously had a haemorrhagic stroke within his left thalamus (Fig. 2), resulting in right-sided hemiplegia, which improved to normal following months of intense rehabilitation. He had a residual right-sided hemi-sensory loss and a 3–5 Hz action tremor. The risk of haemorrhage from deep CM, of up to 10% per year, has been reported [2, 5]. However, a prior haemorrhage increases the risk of further haemorrhage to 4.5–30% per year [3]. The decision for microsurgical excision of this CM was based on the significant cumulative lifetime risk in this young patient. Rangel-Castilla and Spetzler divided the thalamus into 6 operative regions; our patient’s lesion does not fully fit any of these, however, within the proximity of the interface between regions 3 and 4 [6]. They recommend either a parieto-occipital transcortical trans-ventricular approach or the anterior contralateral inter-hemispheric transcallosal approach. With the availability of fibre tract imaging and the BrainPath® tubular retractor system (TRS), we favoured a parietal trans-sulcal para-fascicular (PTPF) approach without entering the ventricle. There are no major vascular structures encountered along this route, in comparison to the interhemispheric approach. Furthermore, there is minimal traversal of eloquent white matter bundles (Fig. 3).

## Description of the technique

Following the induction of general anaesthesia, transcranial corkscrew electrodes and peripheral needle electrodes were applied for monitoring the cortico-spinal tract (CST), via motor evoked potentials (MEPs) and the spinothalamic tract via somatosensory evoked potentials (SSEPs), respectively. The patient was then positioned in the right lateral position with the head fixed in a 3-point Mayfield clamp.

**Fig. 4:**
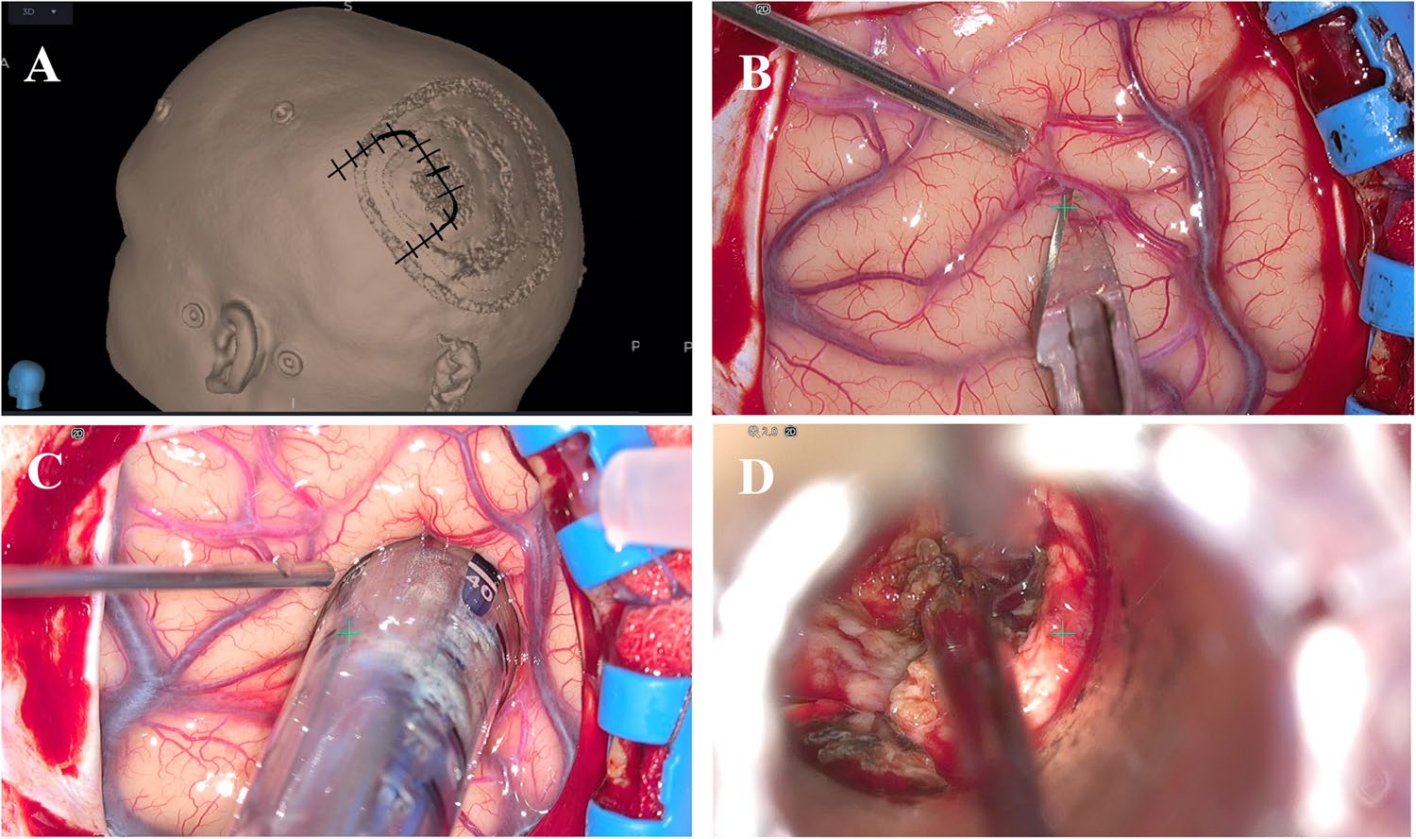
Intraoperative still images of **A** 3D model of patient’s head with horse shoe incision outlined; **B** arachnoidotomy over the intra-parietal sulcus (entry point); **C** BrainPath® insertion in pre-defined trajectory; **D** exoscope view through working port of BrainPath® during piecemeal resection of cavernoma

**Fig. 5:**
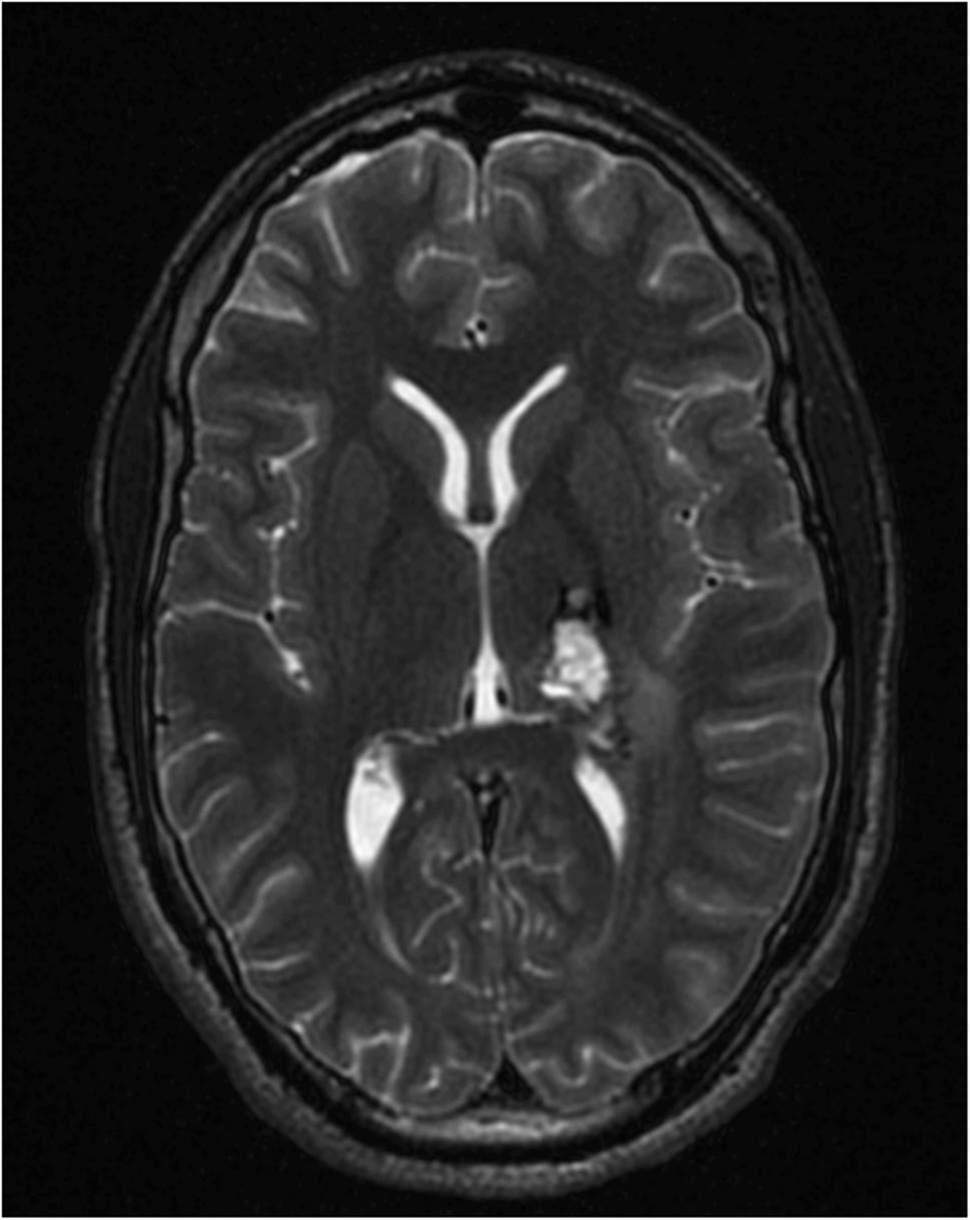
Axial T2-weighted magnetic resonance imaging showing complete resection of cavernoma

A horse-shoe incision was made, followed by a 5-cm parietal craniotomy, allowing adequate exposure around a pre-planned sulcal entry point for an optimal trajectory to the lesion. A curved durotomy was performed to expose the left parietal lobe. The pre-planned sulcal entry point was identified using intra-operative neuro-navigation (Medtronic StealthStation® S8®). A 1-cm linear arachnoidotomy, centred at the desired entry point, was made within the intra-parietal sulcus (operative video). Under continuous image guidance with the neuro-navigation probe in the central channel of the TRS, the BrainPath® obturator (13 mm diameter and 7.5 cm length) was slowly introduced into the sulcus and advanced in the planned trajectory towards the lesion, with constant saline irrigation of the TRS/brain interface (Fig. 4 and operative video). Free electromyography (EMG) recording was performed during this to monitor any triggered responses. The obturator was removed from the external sheath once the target was reached and the TRS was secured in place using the Shepherd’s Hook®. An ORBEYE® exoscope was introduced for magnification and illumination through the working port of the TRS. Direct subcortical monopolar stimulation was performed in order to identify the location of the CST within the IC at the antero-lateral aspect of the CM. A piece-meal resection of the CM was performed, while remaining medial to the IC. Repeat MEPs and SSEPs at the end of the resection revealed no changes from baseline. Haemostasis was achieved and closure performed in the standard fashion. Immediately postoperatively, the patient had a dense right-sided hemiplegia and expressive aphasia. This resolved gradually over the following 72 h. The patient was engaged in intensive physiotherapy over a 2-week period with progressive improvement in his proprioception and somatosensory deficits. Postoperative imaging (Fig. 5) confirms complete resection of the CM.

## Indications

This approach and technique can be considered for lesions located in the posterolateral thalamus/posterior internal capsule. This location is slightly more lateral compared to regions 3 and 4, and slightly more anterior compared to region 5 of the thalamus as described by Rangel-Castilla and Spetzler [6]. The extension of this CM towards the lateral thalamus/internal capsule interface slightly displaces the descending CST fibres and ascending superior thalamo-cortical radiation. This creates a slightly wider entry window lateral to the atrium and temporal horn of the lateral ventricle. This trajectory is favourable for lesions in this region due to the avoidance of commissural fibres, ventricle, and basal ganglia when compared to previously described para-fascicular approaches [1, 7]. As the CST fibres were located anterior and lateral to the CM (Fig. 3C), resection of the lesion through a posterior trans-Sylvian approach was not chosen [4].

## Limitations

The para-fascicular route to lesions in this region might only be feasible with the availability of a TRS such as the NICO BrainPath® allowing atraumatic passage around fibre tracts. Such a system allows rapid accurate placement of the TRS through a safe surgical pathway determined by fibre tractography. We do not recommend the use of this trajectory with the traditional microsurgical entry and advancement of non-navigable retractor blades because of risk of injury to the surrounding white matter tracts. This approach may not be suitable for lesions located centrally within the thalamus, as this would involve traversing the ventral lateral and ventral posterior nuclei of the thalamus. The suitability of other approaches mentioned earlier should be meticulously considered pre-operatively.

## How I avoid complications

Complication avoidance is achieved through a number of factors, starting with precise pre-operative image interpretation and trajectory planning. White matter tractography is a necessity for this approach in order to preserve the integrity of eloquent axonal tracts, particularly, corticospinal tract, arcuate fasciculus, and the optic radiation. Secondly, although surface optical registration is very accurate for neuro-navigation, the senior author prefers fiducials for high fidelity cases such as this. Intraoperative neurophysiological monitoring adds information to that obtained from preoperative tractography, and gives real-time information to the neurosurgeon. During the resection through the tubular retractor, remain within the haemosiderin lined cavity to avoid transgression into nearby white matter fibres, particularly, the corticospinal tract anteriorly within the IC. Meticulous haemostasis is necessary to avoid postoperative haemorrhage.

## Specific perioperative considerations

Ample time must be dedicated to preoperative imaging interpretation and meticulous planning. Perioperative lumbar drainage is not advised for this approach as brain shift from cerebrospinal fluid drainage can alter the accuracy of the navigation system. Patients should ideally be evaluated before and after treatment for language and cognitive function, as well as visual field analysis.

## Specific information to give to the patient about surgery and potential risks

Preoperatively, all management options including conservative management and stereotactic radiosurgery should be discussed with the patient. Available surgical trajectories and associated risks should also be discussed. The risks to speech, contralateral motor function, and visual fields should be adequately explained. The patient should be informed and prepared for postoperative hemiplegia due to manipulation around the CST which in the majority of patients is transient.

## Supplementary Information

Below is the link to the electronic supplementary material.

Supplementary file1 (MP4 410217 KB)
